# Treating the dead; how far ought medicine go to obtain transplantable organs?

**DOI:** 10.3389/frtra.2023.1297957

**Published:** 2023-11-17

**Authors:** Joshua D. Bernstock, Joshua I. Chalif, Rohan Jha, Ashley Brown, Walid I. Essayed, Arthur Caplan, Pierpaolo Peruzzi

**Affiliations:** ^1^ Department of Neurosurgery, Brigham and Women’s Hospital, Harvard Medical School, Boston, MA, United States; ^2^ Division of Medical Ethics, Grossman School of Medicine, New York University, New York, NY, United States

**Keywords:** brain death, transplantation, organ donation, ethics, medical resource utilization

## Abstract

Under what circumstances, is it ethical to perform tumor surgery on a brain-dead individual? The neurosurgeons at Brigham and Women's Hospital were recently faced with such a question when asked to operate on a 28-year-old man who was pronounced brain-dead secondary to a severe brain-stem injury. His advanced directives clearly documented a desire for organ donation. During his transplant work-up, cranial imaging suggested a possible cerebellar mass of unknown etiology that was concerning for metastatic disease. Despite negative full body imaging, the neurosurgical team was asked to perform an open biopsy of the intracranial lesion to rule out occult systemic cancer. This case invites many nuanced questions related to the decisions surgeons and the broader medical community must make in the face of pursuing viable organs for the many in need. What is the moral standing and personhood eligibility of brain-dead individuals? What is the scope of medical interventions and procedures that surgeons are ethically bound to carry out? How ought the desire for increased medical intervention to try to save organs be balanced with practical limitations given limited medical resources?

In 2021 there were over 105,000 individuals on the transplant waiting list, whereas only 40,000 transplants were performed (1). Dead and brain-dead individuals comprise the primary sources of these organs, but their donation is limited by three factors: family refusal, hemodynamic collapse after brain death, and/or exclusion criteria (2); the latter include but are not limited to active malignancy, HIV (although this is changing), and/or evidence of acute/active infection (3). Of donors with a cancer history, the second most common cancer was brain tumors (4). As a result, many individuals are not able to fulfill their posthumous wish of becoming organ donors, with recent estimates suggesting up to 30% of eligible donors are ultimately screened out (5).

Death itself over time has emerged from the concept of irreversible cessation of cardiovascular function to the irreversible loss of brain function(s) (2). In the United States, there is no uniformity to certify brain death, but the medical community generally accepts the American Academy of Neurology's position statement, which endorses brain death as complete loss of consciousness, brainstem reflexes, and ventilatory drive in “absence of factors that imply possible reversibility” (6). Imaging correlates such as a lack of cerebral blood flow are often used to compliment the clinical diagnosis defined above (7). However, given medical advances such as ventilation and ECMO, medicine intervenes in the trajectory that once usually defined death and as a result, increasingly, the burden falls on the provider to ascertain when death has occurred (8). As a natural consequence, what it means to be a human person, i.e., what it means to be irreducibly whole and have incommensurable value, becomes a physiological question, instead of an ontological one.

Given that living human beings are afforded an ethically inviolable moral status and personhood, what moral right should brain-dead individuals be given, if any? The term “moral status” is used in ethics and philosophy to refer to the intrinsic value of particular entities, and a clear distinction should be made between those living and deceased (9). Those who are deceased should however be respected, as they are a symbol of humanity; such respect is a hallmark of civilized society. As there is direct continuity, the deceased should be treated with respect for the values, dignity, and preservation of life's goals of the previously living (10). However, does this respect necessarily imply the preservation of their prior alive self's autonomy, the ability to be provided beneficence, or even be harmed?

Four widely accepted core values of medical ethics are beneficence, non-maleficence, autonomy, and justice (11). The nature of brain death forces a perpetual lack of autonomy. As a result, some contend a brain-dead person's autonomy cannot be violated (12). However, depending on the previously held personal values of the patient, especially regarding the meaning of the body posthumously, some authors maintain brain-dead individuals have “the right of bodily integrity and… precedent autonomy over incompetence-surviving investment interests” (7).

Can benefits be provided to the dead? Clearly, any action carried out when brain-dead will not provide any direct benefits to the individual. If it was an individual's prior wish to become an organ donor, the only direct benefits afforded are to others receiving the life-saving transplant. However, indirect beneficence could be provided by respecting the wishes of the deceased. If it was a patient's wish to perform a highly beneficial act at the end of their life, then ensuring its accomplishment is the only left-over vested best interest of the deceased. To the individual, as there are no competing beneficence interests, could ensuring organ transplantation serve as an effective initial guiding ethical principle? Truly, it would be ethically wrong if the opportunity for organ donation was lost.

However, this pursuit should be tempered by non-maleficence, if pertinent. It begs the question; can brain-dead individuals suffer harm? It is obvious that pain, suffering, and worsening of morbidity and mortality in the conventional sense are not directly possible in the brain-dead state. When individuals consent to organ donation, they are likely consenting under their previously held notion of death, where they have nothing to lose. However, initiation of surgery or other interventions while brain-dead offer two possible sources of harm: disfigurement and the possibility of hemodynamic instability. Yet, patients already implicitly consent to surgical disfigurement in the process of organ retrieval. If one extends that additional procedures and interventions are simply extended means to an end of the same goal, then any degree of justifiable disfigurement to achieving that end is legitimate, and hence there is no additional maleficence.

Nevertheless, brain death is a condition with a non-negligible risk of cardiovascular collapse and hemodynamic instability (13). Surgery likely exacerbates this risk, and if major ischemic events and instability were to occur, the individual's organs are at risk of donation ineligibility. However, if deterioration were to occur, it places the individual in the same state prior to surgery, where they are deceased and their organs ineligible for donation; as a result, non-maleficence is likely maintained. The capability of harm in a brain-dead state continues to be discussed by some (7, 9); however, when interventions are carried out directly in pursuit of ensuring post-humous goals with appropriate respect for the deceased, maleficence is not introduced.

If an individual has chosen to be an organ donor, given the only source of beneficence and lack of maleficence, it is reasonable to ascertain that ethically, the only vested interest they have while brain-dead is to serve as an organ donor? As a result, regardless of the means, should all actions and interventions that facilitate organ retrieval in the brain-death state be considered ethical due diligence? If so, to what extent should this goal be pursued?

In the literature, multiple different interventions have been afforded to brain-dead individuals to ensure eligibility for organ donation. Brain-dead women who were found to be pregnant were maintained for months on a ventilator, before delivering a neonate, and then subsequently undergoing organ donation procedures (14). Individuals have undergone dermatologic evaluation and biopsy to rule out melanoma (15). Moreover, ECMO has been used for preserving organs (13). These resuscitative measures are explicitly not used to save a patient's life, but for hemodynamic stabilization and delaying decisions regarding donor suitability (13). In the pursuit of maximizing organ donation eligibility, multiple invasive diagnostic and therapeutic interventions have been attempted and ethically justified. However, does a practical limit concerning medical resource utilization and futility exist in pursuit of these goals?

Hypothetically, if an early-stage tumor was discovered, in which an alive individual was medically eligible to achieve resection and chemotherapy/radiation, should a similar course be carried out on a brain-dead individual? Under our operating ethical guidance, it falls within the domain of interventions in pursuit of facilitating organ retrieval. If surgery, ECMO, and even facilitating a pregnancy to induction have been within reasonable scope thus far, why would treating tumors not be in scope to ensure the deceased is not screened out by the exclusion criteria? Moreover, active infection is another source of exclusion. If treatment with a source course of antibiotics is otherwise indicated, why not facilitate this treatment? Does our hesitancy to go forth with these avenues stem from our discomfort with the increasing role of instrumentalization of brain death? Or is it from medical resource futility, where all these measures become too much of a burden on health care resources?

When an individual is alive, medical futility is when treatment is not likely to benefit the patient. Consequences include patient suffering, physician distress, financial burdens, and healthcare resource diversion (16). With respect to organ transplantation from brain-dead individuals, the potential benefits to the recipients are immense. However, the scope of interventions comes directly from the opportunity cost of treating other individuals. In well-resourced areas, it is of less concern, but justice requires the fair and equitable distribution of resources. Every antibiotic course, ventilator and ECMO use, ICU bedding and staffing, and surgical procedure has an opportunity cost, as well as an economic cost. In addition, there is a question of who should decide whether interventions to facilitate organ donation are excessive, with respect to opportunity or actual cost. Should it be the provider asked to perform the procedure? Or should it be the surrogate decision maker, who is then put in the precarious position of being the reason for violating whatever “precedent autonomy” has guided the pursuit of organ donation up until a point. Or yet, should it be other third-party stakeholders?

Complicating this matter is that successful organ procurement is never guaranteed with these interventions. An alive, savable patient who needs these interventions should be given priority. As a result, the time course and complexity of interventions, their scarcity, as well as their role in progressing donation must be weighed. Similarly, the number of organs that can be retrieved should be factored in as well, as it likely correlates with the number of distinct lives that can be impacted/saved. Some authors recommend interventions enabling donations to be limited to 24 h, though other have suggested longer timeframes for procurement (17, 18). Given the unmet needs of organs, it seems reasonable to possibly extend this time frame, but the question remains for how long and under what resource utilization circumstances. There likely is a point where the marginal gain from transplantation is significantly diminishing with respect to resource utilization. However, an earnest evaluation would require analysis of context, resource availability at the donor hospital, outside hospital transfer demands, blood or medication shortages, or extenuating circumstances such as ongoing pandemics.

If a brain-dead individual had chosen to be an organ donor, then all procedures to this means seem ethically valid with respect to the donor. However, the opportunity cost of medical resource utilization serves as ethical constraint with respect to the broader medical community. It is nonetheless time to reach consensus as to when it is appropriate to intervene, resources permitting, to ‘treat’ the dead to assist the living who need transplants.
